# Don't Forget About New Pathology

**DOI:** 10.7759/cureus.56080

**Published:** 2024-03-13

**Authors:** Tasciana T Gordon, Tony Mallett

**Affiliations:** 1 General Surgery, Mater Hospital, Brisbane, AUS

**Keywords:** intra-abdominal collection, surgical complication, metastatic melanoma, acute complicated appendicitis, appendicitis

## Abstract

Appendicitis is an inflammatory condition of the appendix. Patients typically present with migratory right iliac fossa pain, reduced appetite, fever, nausea and vomiting. Despite its characteristic presentation, diagnosis remains challenging, particularly in cases where there has been unrelated prior surgery which may obscure the clinical picture. We present a case of a 59-year-old male who had three previous needle aspirations following a pelvic and inguinal lymph node dissection for metastatic melanoma subsequently presenting with a further episode of right iliac fossa pain. This case underscores the diagnostic challenges that may arise in individuals with a history of surgical interventions, emphasizing the importance of a comprehensive approach to ensure the timely and accurate identification of appendicitis.

## Introduction

In the realm of clinical presentations, appendicitis stands as a commonplace yet diagnostically intricate challenge. Appendicitis is typically characterized by migratory right iliac fossa pain, diminished appetite, fevers, nausea and vomiting [1]. However, its diagnosis becomes particularly complex when obscured by prior surgeries or other comorbidities such as metastatic melanoma, leading to potential pitfalls in clinical assessment. This is exemplified in the case we present: a 59-year-old male with a noteworthy medical history, including three prior needle aspirations and an inguinal and pelvic lymph node dissection for metastatic melanoma. This case underscores the need for a comprehensive clinical acumen that ensures new pathology is considered in the differential diagnosis, especially in patients with a history of surgical intervention and metastatic melanoma.

## Case presentation

**Figure 1:**
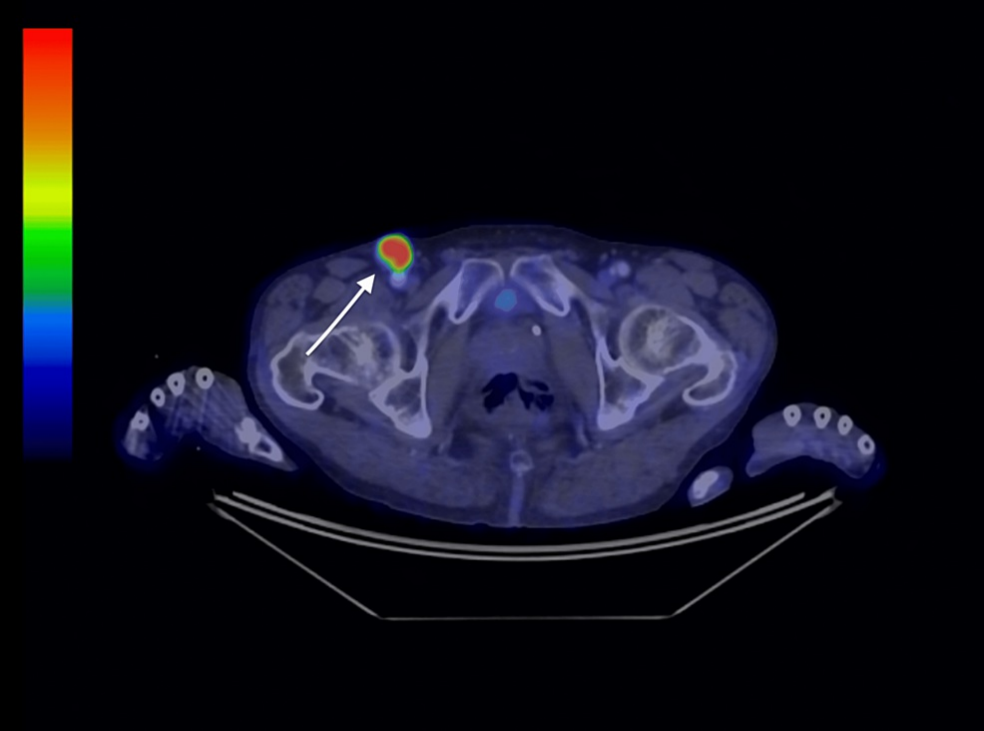
PET CT showing a right inguinal lymph node indicated by a white arrow

The patient is a 59-year-old male who five months prior to admission presented to his GP with groin lumps and unintentional weight loss over 18 months. The GP arranged a PET CT which showed three enlarged right inguinal lymph nodes measuring up to 24x22mm with intense FDG avidity (see Figure 1). There were no other skin lesions or lymphadenopathy identified elsewhere. The patient subsequently underwent a right groin lymph node biopsy which confirmed metastatic melanoma, BRAF wild type, SOX 10+, S100+.

The case was discussed at the melanoma multidisciplinary team meeting. The recommendation was to progress to a right pelvic and inguinal lymph node dissection. Histology showed 3/13 involved inguinal nodes with pelvic sidewall and external iliac nodes 0/8, 0/6 respectively. The patient was subsequently commenced on nivolumab.

Pathology demonstrated stage IIIB/C (TxN2bM0) BRAF wild-type melanoma of the right groin and was diagnosed with metastatic melanoma of unknown primary location.

There was no family history of melanoma. However, there was a family history of breast and ovarian cancer (mother and maternal aunt had breast cancer and paternal aunt and paternal grandmother had ovarian cancer). He underwent basal cell carcinoma excisions from the right deltoid and right scapula in 2020. Margins were clear. He is also a current smoker (seven cigarettes daily). 

Post-operatively, the surgery was complicated by an inguinal seroma. This was aspirated in a clinic setting via palpation with a needle on three separate occasions. The patient was also advised to wear compressive garments to assist with reducing the risk of recollection.

Four months following the pelvic and inguinal dissection and one month following the last needle aspiration, the patient presented to the emergency department with right lower quadrant pain. He reported a reduced appetite and feeling generally unwell. On examination, his abdomen was soft, he was tender in the right iliac fossa and suprapubic region. There was no rebound tenderness or concerns of peritonism. There were also no skin changes at the surgical site suggestive of a surgical site infection. Pathology showed an elevated white cell count of 21.2x10^9/L (4.0-11.0) with neutrophilia 17.8x10^9/L (1.8-7.7) and an elevated C-reactive protein 195mg/L (<6) (see Table 1).

**Table 1:** Pathology results on presentation *H = high; x10^9/L =  result x 1 000 000 000 (one billion) cells per litre; x10^12/L = result x 1 000 000 000 000 (one trillion) cells per litre; g/L = grams per litre; L/L= litre of red blood cells per litre of blood; fL = femtolitre; pg = picogram; % = percentage; mg/L = milligram per litre

Blood Count	Results	Units	Reference Ranges
White cell count	21.2H	x10^9/L	4.0-11.0
Haemoglobin	130	g/L	130-180
Platelets	688H	x10^9/L	150-450
Haematocrit	0.387	L/L	0.39-0.52
Red cell count	4.10	x10^12/L	4.5-6.0
Mean cell volume	94.4	fL	80-100
Mean haemoglobin concentration	31.7	pg	27.0-33.0
Mean corpuscular haemoglobin concentration	336	-	310-365
Red cell distribution width	12.7	x10^9/L	<16.5
White cell differentiation			
Neutrophils	17.80H	x10^9/L	1.8-7.7
Lymphocytes	1.02	x10^9/L	1.0-4.0
Monocytes	2.14H	x10^9/L	0.2-1.0
Eosinophils	0.04	x10^9/L	0.04-0.5
Basophils	0.04	x10^9/L	<0.15
Left shift	0.15	x10^9/L	
Left shift %	0.7	-	<1.0
CRP	195	mg/L	<10

**Figure 2:**
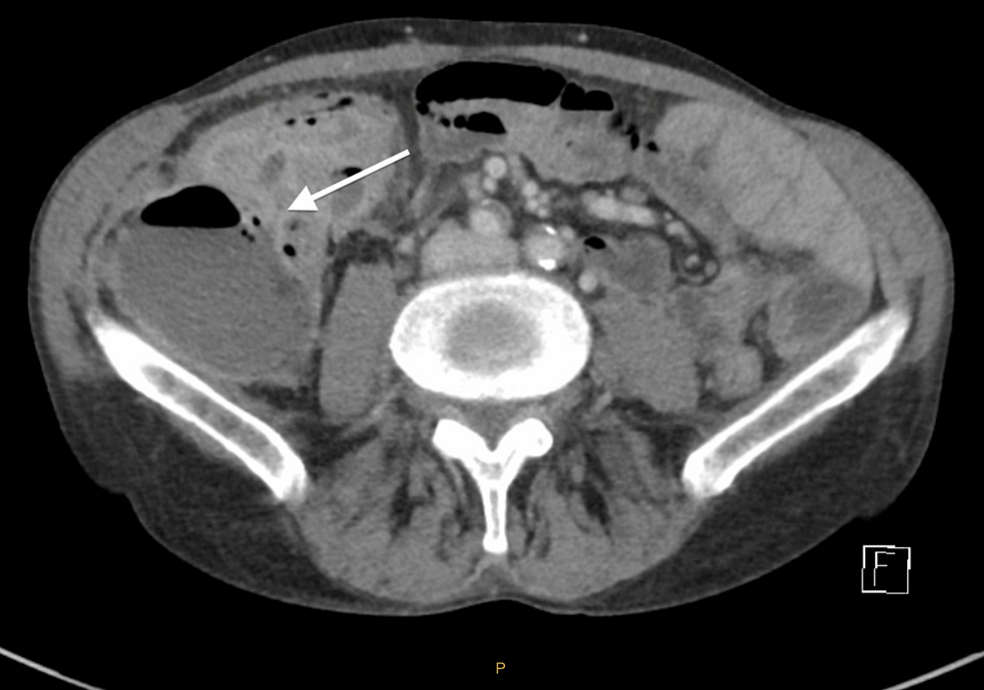
CT abdomen showing intra-abdominal collection marked by a white arrow

Computed tomography (CT) abdomen showed a collection in the right lower quadrant abutting the right psoas muscle (see Figure 2). It was initially thought that this may be a post-operative abscess, bowel perforation, or recurrence of metastatic melanoma. However, a recent PET CT completed six weeks earlier showed no signs of recurrent disease so this was thought to be less likely. New pathology such as appendicitis was not considered in the initial review.

**Figure 3:**
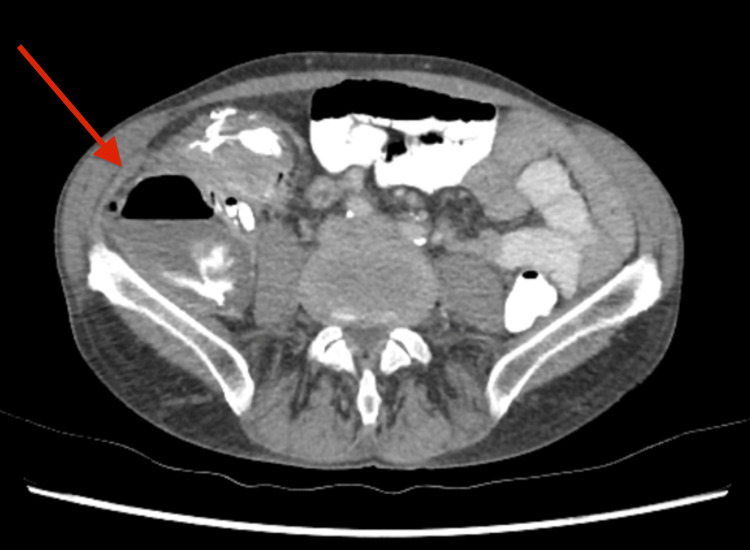
CT abdomen with oral contrast demonstrating extravasation of contrast into intra-abdominal collection

Oral contrast CT was performed and showed contrast extravasating into this collection (see Figure 3). This then raised the concern of the collection communicating with the bowel, confirming the suspicion of bowel perforation. After discussion with our radiologists, we were suspicious of perforated appendicitis as there was an extension of contrast filling a narrow tube attached to the caecum and then spilling into the collection. The decision was to proceed with a diagnostic laparoscopy compared with the radiological aspiration of the collection.

**Figure 4:**
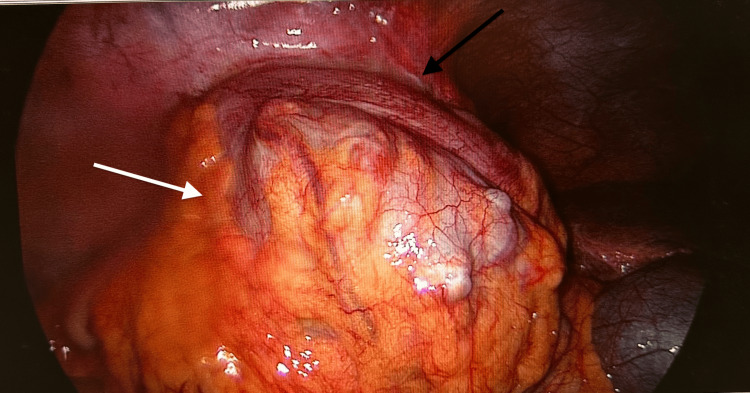
Laparoscopic image with the white arrow indicating collection and the black arrow indicating appendix

**Figure 5:**
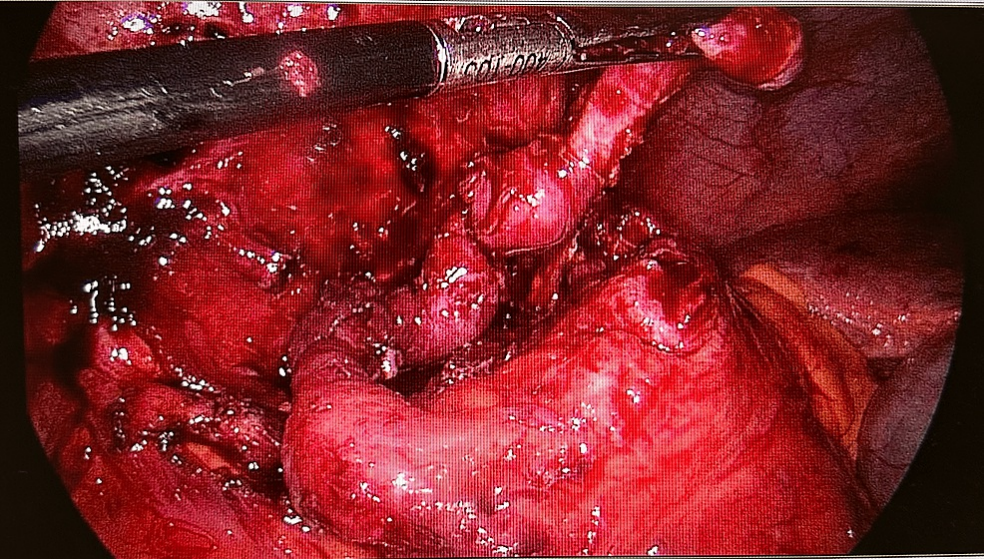
Laparoscopic image demonstrating the inflamed appendix within the laparoscopic instrument

Diagnostic laparoscopy showed a 32x54mm appendiceal collection (see Figure 4). The collection was washed out and an appendicectomy was completed (see Figure 5). The histology showed diverticulosis of the appendix with numerous diverticula identified. There were subserosal fibrosis, fat necrosis, and inflammatory infiltration consistent with appendicitis. He was treated with piperacillin and tazobactam (Piptaz®) for five days. The patient was discharged on day six and was recommenced on nivolumab two weeks later.

## Discussion

Appendicitis is a common presentation managed by general surgeons. The diagnosis of appendicitis can be obscured due to a history of recent previous intra-abdominal surgery at the same site. In order to accurately diagnose patients, it is important to consider new pathology when assessing a patient and not assume that it is related to a previous procedure or comorbidity. Having that index of suspicion will ensure the diagnosis isn't missed. The Alvarado score and utilisation of imaging can also be implemented in these situations of uncertainty. 

Utilising the Alvarado scoring system can assist with identifying those at high risk of acute appendicitis. However, it should be used in conjunction with other modalities such as imaging. The sensitivity and specificity are 66.4% and 69.8% respectively as reported by Al-Tarakji et al. [1]. 

The sensitivity and specificity of CT for the diagnosis of appendicitis in adults are high [2]. Unenhanced standard-dose CT appears to have lower sensitivity than standard-dose CT with intravenous, rectal, or oral contrast. Sensitivity has been reported as 95% with a specificity of 94% [2]. 

Groin and pelvic dissections, while effective in managing metastatic melanoma as in this case, may be associated with certain complications. One common complication is the formation of collections, such as hematomas or seromas [3]. Seromas result from the accumulation of serous fluid at the surgical site and may contribute to delayed wound healing [3]. Both complications, if not appropriately addressed, can increase the risk of infection and hinder the recovery process. In addition to collection-related issues, patients undergoing groin dissections may also experience lymphedema or surgical site infections. There is no evidence of an association between groin dissection and acute appendicitis in the literature. 

Diagnostic laparoscopy enables direct visualization and assessment of the suspected collection in real time. This approach provides a comprehensive examination, allowing for precise identification of tissues, thorough assessment of the appendix, and the potential for immediate intervention if necessary. Diagnostic laparoscopy also allowed for progression to a bowel resection if indicated due to concerns of bowel perforation (contrast leak within the collection). It also allows for conversion to an open procedure if required.

In contrast, radiological aspiration, while less invasive, offers limited visualization and primarily serves a diagnostic purpose without therapeutic intervention [4,5]. There were concerns raised about the development of a chronic fistula and further delay to his adjuvant immunotherapy if we proceeded with a percutaneous drain. 

The choice between these approaches is influenced by factors such as the patient's clinical condition, the urgency of intervention, and the surgeon's preference. This is aimed at ensuring an accurate diagnosis and timely management of appendicitis-related intra-abdominal pelvic collections [4,5]. The evidence of contrast leaking into the collection helped make the decision to proceed to laparoscopy with concerns about bowel perforation. 

At that procedure, the omentum walling off the perforated appendix was separated to isolate the appendix and collection. The capsule of the collection was opened and the inflamed appendix was noted within. There was an air leak within the collection noted by bubbles coming through the saline wash. This was controlled once the appendix base was secured. That enabled the correct procedure of appendicectomy and drainage to be carried out.

The histology showed diverticula of the appendix. However, it did not confirm diverticulitis of the appendix. As with all intestinal diverticula, appendiceal diverticula can be classified as congenital or acquired [6,7]. Congenital form is rare and a true diverticulum, and the more prevalent acquired form is a false diverticulum on the mesenteric border of the appendix. The incidence of diverticula found in appendicectomy specimens ranges from 0.004% to 2.1% [7]. Histological evaluation after appendicectomy is essential to avoid missing other pathologies in the appendix [7]. 

## Conclusions

This case demonstrates that it can not be assumed that what might be thought to be a recurrent problem is necessarily the case. This case highlights the complex diagnostic challenges that individuals with a history of prior surgical interventions may encounter. This underscores the necessity for a thorough approach to ensure the prompt and accurate identification of appendicitis. There needs to be a clinical suspicion for new pathology when seeing these patients. Utilising the Alvarado score as well as imaging may assist with minimising assumptions and allow for an accurate diagnosis. In this case, imaging allowed for the appropriate management with a diagnostic laparoscopy. This allowed visualisation of the tissues to exclude other differential diagnoses and ensured a timely and accurate diagnosis with prompt management to prevent further complications.
